# How Does Physical Activity Intervention Improve Self-Esteem and Self-Concept in Children and Adolescents? Evidence from a Meta-Analysis

**DOI:** 10.1371/journal.pone.0134804

**Published:** 2015-08-04

**Authors:** Mingli Liu, Lang Wu, Qingsen Ming

**Affiliations:** 1 Medical Psychological Institute, Second Xiangya Hospital, Central South University, Changsha, Hunan, 410011, China; 2 School of Education, Hunan University of Science and Technology, Xiangtan, Hunan, 411201, China; 3 Center for Clinical and Translational Science, Mayo Clinic, Rochester, MN, 55905, United States of America; Merced, UNITED STATES

## Abstract

**Objective:**

To perform a systematic review and meta-analysis for the effects of physical activity intervention on self-esteem and self-concept in children and adolescents, and to identify moderator variables by meta-regression.

**Design:**

A meta-analysis and meta-regression.

**Method:**

Relevant studies were identified through a comprehensive search of electronic databases. Study inclusion criteria were: (1) intervention should be supervised physical activity, (2) reported sufficient data to estimate pooled effect sizes of physical activity intervention on self-esteem or self-concept, (3) participants’ ages ranged from 3 to 20 years, and (4) a control or comparison group was included. For each study, study design, intervention design and participant characteristics were extracted. R software (version 3.1.3) and Stata (version 12.0) were used to synthesize effect sizes and perform moderation analyses for determining moderators.

**Results:**

Twenty-five randomized controlled trial (RCT) studies and 13 non-randomized controlled trial (non-RCT) studies including a total of 2991 cases were identified. Significant positive effects were found in RCTs for intervention of physical activity alone on general self outcomes (Hedges’ g = 0.29, 95% confidence interval [CI]: 0.14 to 0.45; p = 0.001), self-concept (Hedges’ g = 0.49, 95%CI: 0.10 to 0.88, p = 0.014) and self-worth (Hedges’ g = 0.31, 95%CI: 0.13 to 0.49, p = 0.005). There was no significant effect of intervention of physical activity alone on any outcomes in non-RCTs, as well as in studies with intervention of physical activity combined with other strategies. Meta-regression analysis revealed that higher treatment effects were associated with setting of intervention in RCTs (β = 0.31, 95%CI: 0.07 to 0.55, p = 0.013).

**Conclusion:**

Intervention of physical activity alone is associated with increased self-concept and self-worth in children and adolescents. And there is a stronger association with school-based and gymnasium-based intervention compared with other settings.

## Introduction

Mental health problems cause huge public health burden in juveniles globally, as evidenced by a 20% prevalence [1, 2]. Research has demonstrated that physical activity (PA) may provide physiological and psychological benefits[3, 4]. Compared with traditional interventions, such as psychotherapy, psychosocial, and pharmacological interventions, PA has few side effects and is relatively cost-effective. Moreover, PA can be self-sustaining[5]. Besides the beneficial effects of PA on cardiovascular disease, diabetes, hypertension, cancer, osteoporosis and obesity[6–8], a growing literature suggests that PA can improve mental health[8, 9], including depression, anxiety, self-esteem (SE), self-concept(SC), anger, stress, executive function and so on[10–13].

SE is defined as feelings of one’s personal self-worth (SW)[14], reflecting person’s evaluation of his or her own worth. And SC is a person’s perceptions of himself or herself, namely, what a person thinks about himself [15, 16]. They both have pervasive impact on human mental status and behavior[17, 18]. Positive SC is viewed as a desirable outcome in many educational and psychological situations, and SC is regarded as a mediating variable for promoting the achievement of certain outcomes, such as academic achievement[19]. Furthermore, physical SC is suggested to be a mediator of the association between PA and SE, which is inversely related to depression [4]. SE has been recognized as a component of a variety of psychopathologies. A search of the DSM-IV-TR [20] shows that the term "self-esteem" appears in 24 different diagnostic contexts as a criterion for disorders. For teenagers, it is suggested that low SE predicts adolescents’ report of mental status and health compromising behaviors, such as depression, anxiety, problem in eating and suicidal ideation [21–23]. Low level of SE in children and adolescents also predicts poor health, criminal behavior, and limited economic prospects during adulthood[24, 25]. Thus it is important to determine effective interventions for improving SE and SC for juveniles.

Despite that extensive research has evaluated the effects of PA on SE and SC in juveniles, contradictory findings have been suggested. Although many studies found that there were significant positive effects of PA on SE and SC[13, 26, 27], others did not detect such effects[28–30], let alone several others suggested negative effects[31–33]. Therefore, it is critical to comprehensively synthesize available evidence to determine the exact effects of PA on SE and SC in children and adolescents. Besides, whether the effects of PA intervention on SE and SC are context-dependent by moderators should be clarified to reveal in which conditions the effects exist. Meta-analysis of all available evidence is an appropriate design to clarify these questions.

Two meta-analyses have partially evaluated these questions. The study by Ekeland and colleagues (2005) identified moderate positive effects of PA alone (SMD = 0.49, 95% CI: 0.16, 0.81) and PA combining with other skills (SMD = 0.51, 95%CI: 0.15, 0.88) on SE[34]. Another study by Ahn and colleagues (2010) suggested there were significant effects of PA on SE ranging from slightly in randomized controlled trails (RCTs) (Hedges’ g = 0.29, SE = 0.08) to large in non-randomized controlled trials (non-RCTs) (Hedges’ g = 0.78, SE = 0.28); and the effect of PA on SC was small in RCTs (Hedges’ g = 0.16, SE = 0.10). However, there was no convincing evidence to support PA intervention’s beneficial effect on SC in non-RCTs (Hedges’ g = 0.12, SE = 0.31) and on SE in correlation studies (average correlation coefficient r = 0.04, SE = 0.04)[35].

Despite the overall synthesization, there are several limitations for these studies. The majority of included studies in Ekland’s meta-analysis suffered from both high risk of bias and small sample size. On the other hand, as Dishman suggested, it is warranted to pay attention to important moderator variables to better clarify the research questions[36]. Although Ekland and colleagues examined a potential moderator (study quality) of the association between PA and SE, the evaluation is compromised by the limited number of studies involved in the subgroups [34]. Additionally, neither of the two meta-analyses specially explored any other potential moderators of PA intervention on SE or SC, such as participant type, intervention setting, and so on.

Since the meta-analysis conducted by Ahn et al, eleven trials examining the effects of PA on SE or SC have been published. The availability of these studies makes it possible to perform more comprehensive meta-regression analyses to identify additional moderators. Therefore, it is necessary to conduct an updated meta-analysis to provide a more accurate estimation for these research questions.

The purpose of the present study was thus to perform a meta-analysis of available literature to evaluate the efficacy of PA intervention on SE and SC in juveniles, and conduct a meta-regression analysis to identify effect moderators. We aimed to figure out whether PA intervention might exert positive effects, and in which participants and settings the positive effect persist. Based on dose-response models[36, 37] and relevant meta-analysis[3], we examined several potential moderators by meta-regression analysis, including: target population, PA setting and PA characteristics (intensity per session, frequency and length of intervention), and study quality.

## Methods

### Selection of study

We followed the PRISMA guidelines[38] to report this systematic review and meta-analysis (S1 File). The electronic databases of PubMed, EBSCO, and Web of Science (up to July 2014) were searched for RCTs or non-RCTs in children and adolescents without restriction of population, publication type, and language. The following MeSH terms and their combinations of the Title/Abstract/Subject were used in the search: physical activity / exercise / sport*; self esteem* / self worth* /self concept* /self perception*; children / adolescent* / boy* / girl* / teen*; random* / intervention* / trial*. The asterisk means that larger words that contained the word or word fragment were included in the search. Furthermore, the reference lists of eligible articles were scrutinized by hand to identify additional studies.

The studies were included when the following inclusion criteria were met: (1) intervention should be supervised PA or PA combined with other strategies; (2) reported sufficient data to estimate pooled effect sizes of PA intervention on SE or SC; (3) sample participants’ age ranged from 3 to 20 years; (4) included a non-PA control or comparison group. When multiple reports representing the same study were found, the most relevant or complete report was included. Reports stratified by gender were treated as separate reports. Owing to the equating of the concept and operation of SE, SC, and SW in various studies[13, 39, 40], we took all three outcome measurements into account and evaluated their benefits from PA intervention. Only the most relevant self outcome type was included in analysis.

### Statistical methods

Relevant data from the included studies were extracted independently by two authors using EpiData 3.1 and Excel software. The following information was extracted from each study: first author, year, study design, participant characteristics, outcome measure, PA intervention design and effect sizes. Any disagreements were discussed until consensus was reached. When the eligible studies did not present sufficient data, corresponding or first authors were contacted.

Study quality was assessed using the modified Cochrane risk of bias tool[41] for RCTs and the modified Methodological Index for Non-Randomized Studies (MINORS)[42] for non-RCTs. The former consists of seven items: randomization sequence generation, inclusion and exclusion criteria, balance between groups at baseline, allocation concealment, blinding of participants, dropout and withdrawals, and follow up. A score of 1 was given for each of the points described above. Higher scores indicate higher study quality. The quality scale ranges from 0 to 7 points. Studies achieved ≥ 5 points were considered to be with high quality. The MINORS includes twelve items: stated aim of the study, inclusion of consecutive participants, prospective collection of data, endpoint appropriate to the study aim, unbiased evaluation of endpoints, follow-up period appropriate to the major endpoint, loss to follow up not exceeding 5%, a control group having the gold standard intervention, contemporary groups, baseline equivalence of the sample size, and statistical analyses adapted to the study design. Scoring formula was similar to RCTs’. And studies achieved ≥ 6 points were considered to be with high quality.

Subtypes were coded separately by two authors. There were three primary classifications: study design, PA intervention, and participant characteristics. Study design was coded according to research design (RCT or non-RCT), outcome measure (SE, SC, SW), and study quality (score ≥ 5 or score < 5 in RCTs; score ≥ 6 or score < 6 in Non-RCTs). PA intervention was coded by PA intervention type (PA alone or PA combined with other skills), intervention setting (school-based, gymnasium-based, family-based, clinic-based, detention facility-based, or camp-based), intensity of PA intervention (minutes per session), frequency (times per week) (1 or 2 or 3 or 4 or 5 or 6), and length of intervention (weeks). Participant characteristics consisted of gender (female or male), and sample type (normal, overweight, cerebral palsy, youth offender, sedentary, disability, or asthma).

We used R software (version 3.1.3) and Stata (version 12.0) to conduct all statistical analyses. Hedges adjusted *g* was used as a measure of effect size because of its unbiased properties for small sample sizes compared with Cohen’s *d*[43]. The effect size of each included study was calculated by computing the mean difference in gains (posttest–pretest) between the intervention group and control group and dividing by the pooled standard deviations of pre-test scores[44], since pretest standard deviations will not be influenced by different treatments and thus tend to be consistent across studies[45]. For pooling estimates, we combined the data on PA intervention outcomes from the included studies using the random effects model with weighing each effect size estimated by the DerSimonian & Laird. When the heterogeneity was zero, the fixed effects model was applied. We converted DerSimonian & Laird results to Hartung and Knapp results when the pooling analysis included more than five studies[46], since in such case the distribution of the intervention effects is unknown and it does not necessarily follow the normal or t-distribution when study numbers are small[46]. All analysis results were reported with 95% confidence intervals (CIs). And statistical significance was defined as p < .05.

To determine pooled effect sizes for different subgroups according to subtype of outcome measurement, PA intervention, and study design, we conducted separate meta-analysis for subtype of PA intervention (PA alone or PA combined with other strategies), in combination with subtype of study design (RCTs or nor-RCTs) and outcome measure (SE, SC, or SW).

Heterogeneity of effect size was assessed using the Q value and I-square statistic. To explore sources of heterogeneity, we performed planned meta-regression analysis. Based on previous analysis[35], potential between-study moderators were examined, including study quality, intervention format (intensity, frequency, and length), setting of intervention, and participant type. Meta-regression analyses according to subtypes of study design were also conducted.

Additionally, we conducted sensitivity analyses. Outlier was identified if the relative residual standardized mean difference effect size fell in the region of z< -1.96 or z>1.96 by omitting the study. Publication bias was assessed by a funnel plot accompanying with Begg’s test [47]and Egger’s test[48]. Adjusting for publication bias was assessed by the trim and fill method if there was significant publication bias[49].

## Results

### Characteristic of included studies

**Fig 1:**
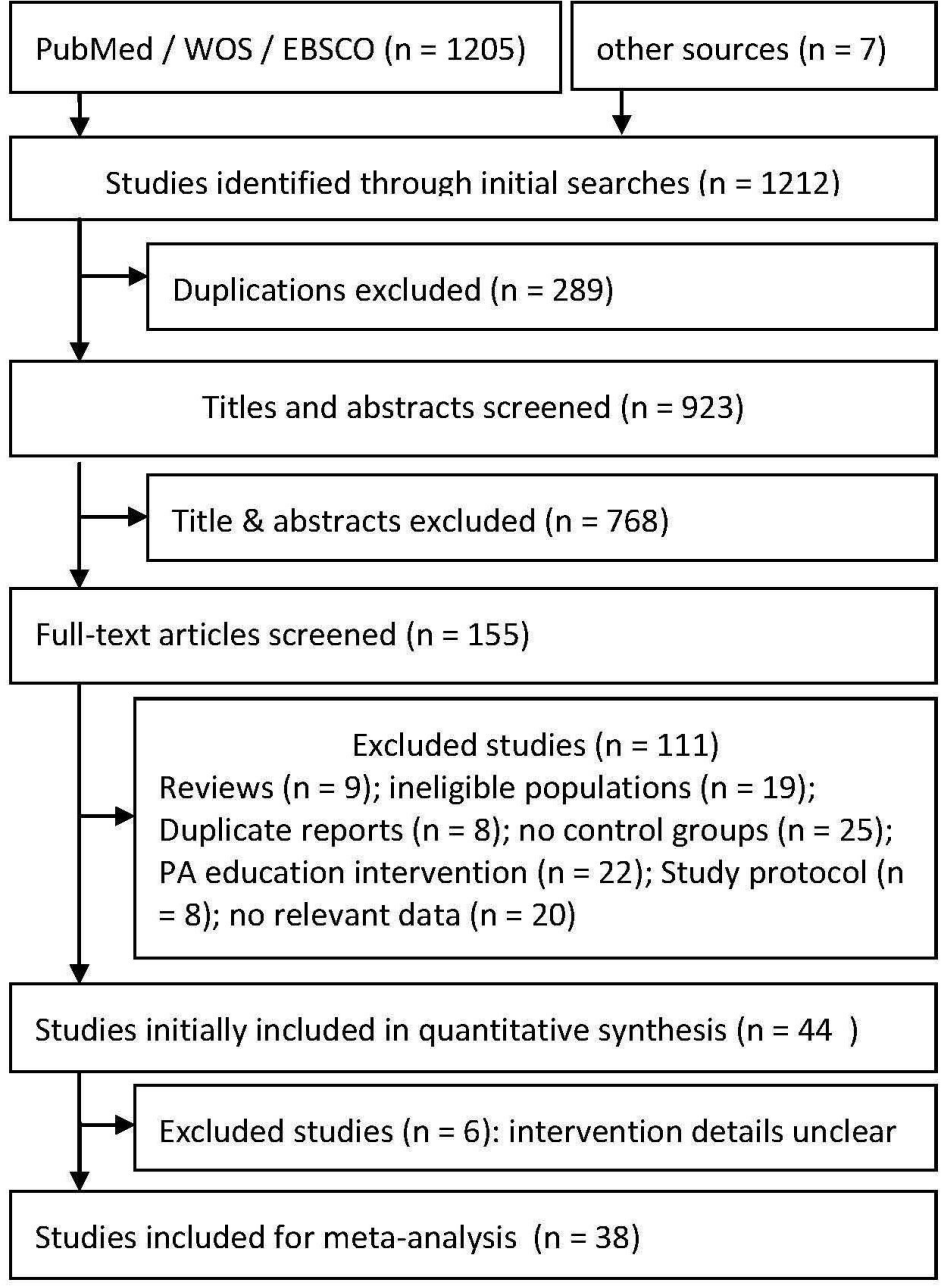
Flow chart of article screening process.

After article screening, 38 studies with 39 reports (one study[50] reported results stratified by gender) including 2991 participants (1683 in treatment group and 1308 in control group) fulfilled the inclusion criteria and were included in the final analysis (Fig 1). Most of included studies were conducted in the United States (N = 18), and others were conducted in various countries including: Australia (N = 4), United Kingdom (N = 3), China (N = 2), Netherlands (N = 2), Sweden (N = 2), Canada (N = 1), Germany (N = 1), Israel (N = 1), Portugal (N = 1), South Africa (N = 1), Spain (N = 1), and Switzerland (N = 1).

Among the included studies, 25 were RCTs and 13 were non-RCTs (Tables 1 and 2). Three assessment variables were used to measure the outcome variable, including SE in 19 studies, SC in 7 studies (1 study used self-image to measure SC), and SW in 12 studies. It is worth noting that, although several studies used self-perception scales, only one subscale (SE, SC or SW) was considered as the outcome in each study. About 16 questionnaires or scales were applied to measure the 3 variables. The most commonly used were Rosenberg Self-Esteem Scale (RSE) (N = 7), Self-Perception Profile for Children (SPPC) (N = 6), Self-Esteem Inventory (SEI) (N = 5), and Piers-Harris Children’s Self-Concept Scale (PHCSCS) (N = 3).

**Table 1:** Characteristics of included randomized control trial studies.

Study: authors (year)	No. participants: mean age ± sd (years); target	Physical activity: style; intensity (min/session) / frequency (times/wk) / length (wks)	Outcome: measurement; timepoint (wks) (wks)	Quality score
Alpert, et al (1990)	24; 3.67; normal	PA alone; 30 / 5 / 8	SC; 0–8	3
Daley, et al (2006)	51; 13.1; overweight	PA alone; 30 / 3 / 8	GSW; 0-8-14-24	6
Dodd, et al (2004)	17; 12.14 ± 2.47; cerebral palsy	PA alone; 3sets / 3 / 6	GSW; 0-6-18	6
Faude, et al (2010)	22; 10.8 ± 1.2; overweight	PA alone; 60 / 3 / 12	SE; 0–12	5
Hilyer, et al (1982)	43; 16.96 ± 0.83; offenders	PA combined; 90 / 3 / 20	SE; 0–20	4
Jelalian, et al (2011)	89; 14.2 ± 0.93; overweight	PA combined; 60 / 1 / 16	GSW; 0-16-48	5
Lindwall, et al (2005)	62; 15.31 ± 1.34; sedentary girls	PA combined; 60 / 2 / 24	PSW; 0–24	6
Lubans, et al (2010)	88; 14.96 ± 0.68; normal	PA alone; 40–50 / 2 / 8	PSW; 0–8	6
MacMahon, et al (1987)	54; 9.7; learning disabilities	PA alone; 25 / 5 / 20	SC; 0–20	4
MacMahon, et al (1988)	69; 16.3; offenders	PA alone; 40 / 3 / 12	SC; 0–12	3
Mazzoni, et al (2009)	18; 8.4 ± 1.7; disability	PA alone; 60 / 1 / 6	SW; 0–6	4
Morgan, et al (2012)	100; 14.3 ± 0.6; sedentary boys	PA combined; 90 / 1 / 10	GSW; 0-12-24	4
Munson, et al (1988)	26; 17.2; offenders	PA alone; 60 / 1 / 10	SE; 0–10	3
Petty, et al (2009)	138; 9.34 ± 1.04; overweight	PA alone; 40 / 1 / 13 (-1.6,+1.6)	SW; 0–13	6
Robinson, et al (2010)	243; 9.4 ± 0.9; normal girls	PA combined; 120 / 5 / 40	SE;0-24-48-72-104	5
Sacher, et al (2010)	81; 10.25 ± 1.3; overweight	PA combined;? / 2 / 21	GSE; 0-6-12	6
Schranz, et al (2013)	49; 14.99 ± 1.49; overweight	PA alone; 75 / 3 / 24	SE; 0-12-24-48	6
Staiano, et al (2013)	33; 15–19; overweight	PA alone; 30–60 / 5 / 20	SE; 0–20	4
Unger, et al (2006)	31; 13–18; cerebral palsy	PA alone; 40–60 / 1–3 / 8	SC; 0–8	6
Van Wely, et al (2013)	45; 9.76 ± 1.66; cerebral palsy	PA combined; 60 / 1–2 / 24	SW; 0-24-48	7
Veldhoven, et al (2001)	47; 10.6 ± 1.19; asthma	PA alone; 60 / 2 / 12	SW; 0–12	4
Velez, et al (2010)	28; 16.14 ± 0.19; normal	PA alone; 35–40 / 1 / 12	GSW; 0–12	3
Wagener, et al (2011)	39; 14 ± 1.66; overweight	PA alone; 40–75 / 3 / 10	SE; 0–10	2
Weintraub, et al (2008)	21; 9.98 ± 0.84; overweight	PA alone; 75 / 3–4 / 24	SE; 0–24	2
Yu, et al (2009)	82; 10.5 ± 1; overweight/obese	PA combined; 75 / 3 / 6	PSC; 0–6	5

RCT, Randomized controlled trial; non, non-RCT; SE, self-esteem; SC, self-concept; SW, self-worth; GSW, global self-worth; PSE, physical self-esteem; PSC, physical self-concept; PSW, physical self-worth

?, not indicated

**Table 2:** Characteristics of included Non-Randomized control trial studies.

Study: Author (year)	No. participants: Mean age ± sd (years); target population	Physical activity: style: duration(min/day) / frequency (times/wk) / period (wks)	Outcome: measurement; timepoint (wks)	Quality score
Annesi, et al (2006)	81; 10.8 ± 1.1; normal	PA combined: 45 / 3 / 12	PSC; 0–12	5
Chang, et al (2013)	67; 7th grade; normal	PA alone: 45 / 1 / 8	SE; 0–8	7
Ford, et al (1989)	108; 19.8; girls	PA alone: 3hr /wk / 8	SE; 0–8	5
Gately, et al (2005)	223; 13.9; overweight	PA combined: 60 / 6 / 2–6	GSW; 0–6	4
Hutzler, et al (1998)	46; 5.7 ± 0.94; cerebral palsy	PA alone: 30 / 3 / 24	SC; 0–24	6
Mayorga, et al (2012)	69; 11.1 ± 0.4; normal	PA alone: 15–35 / 2 / 8	SE; 0–8	6
Munson, et al (1985)	31; 17.31 ± 0.87; offenders	PA combined: 90 / 3 / 7	SE; 0–7	4
Percy, et al (1981)	30; 5–6 grade; normal	PA alone:? / 3 / 7	SE; 0–7	5
Schmidt, et al (2013)	464; 11.95 ± 0.55; normal	PA combined: 20–45 / 2 / 10	SE; 0–10	6
Schneider, et al (2008)	120; 15.02 ± 0.77; sedentary girls	PA combined: 60 / 5 / 36	SC; 0-16-36	8
Seabra, et al (2014)	20; 10.42 ± 1.92; overweight boy	PA alone: 60–90 / 4 / 20	SE; 0–20	5
Terjestam, et al (2010)	119; 13–14; normal	PA alone: 25 / 2 / 8	SE; 0–8	6
Wilson, et al (2005)	44; 11 ± 0.64; normal	PA combined: 60 / 3 / 4	SC; 0–4	4

RCT, Randomized controlled trial; non, non-RCT; SE, self-esteem; SC, self-concept; PSC, physical self-concept; GSW, global self-worth

?, not indicated

Twenty-four studies used intervention of PA alone and other 14 studies used intervention of PA combined with other strategies. Intervention setting varied across studies. Twenty-four studies were school-based, 2 interventions were family-based, 5 interventions were gymnasium-based, 3 interventions were clinic-based, 3 interventions were performed in detention facilities, and 1 study was camp-based. The length of intervention ranged from 4 to about 80 weeks (2 academic years). Among them, 7 interventions lasted less than 8 weeks, 24 interventions lasted between 8 to 20 weeks, and 7 trainings lasted over 20 weeks. Exercise intensity for each session ranged from 20 to about 120 minutes. Only 2 studies were with less than 30 minutes per session; 11 studies took 30–45 minutes per session; 21 interventions took more than 45 minutes per session; and the remaining 4 studies did not indicate the exact time. Most PA interventions were administered 2 times (N = 9), 3 times (N = 16), or 5 times (N = 6) per week. The remaining studies held sessions for either 1 time (N = 5), 4 times (N = 1), or 6 times (N = 1) per week.

Sample sizes of the 38 studies ranged from 17 to 464 (the median sample size was 79). Among the 38 studies, 26 studies involved both male and female participants, 8 with only males, and 4 with only females. Participants’ ages ranged from 4 to 20 years. In most studies participants were either normal population (N = 12) or overweight or obese (N = 12); 4 studies targeted cerebral palsy children; 4 studies focused on youth offenders; 3 studies targeted sedentary children or adolescents; 2 studies were focused on individuals with learning or cognitive disability; and 1 study targeted children with asthma.

Generally, the risks of bias for included RCTs were from moderate to high. Twelve RCTs clearly stated that they used randomization sequence generated via computer. Ten studies reported allocation concealment, 7 studies stated blinding method, and 7 studies indicated the follow-up information. On the other hand, the majority of studies reported inclusion and exclusion criteria of participants (N = 22) and explained the reasons of dropout (N = 21). The detailed description of the characteristics of included RCTs is demonstrated in Table 1. For non-RCTs, the risks of bias for included studies were general low. Scores ranged from 4 to 8 points. The detailed description of the characteristics of included non-RCTs is shown in Table 2.

### Meta-analysis of studies with intervention of PA alone

**Fig 2:**
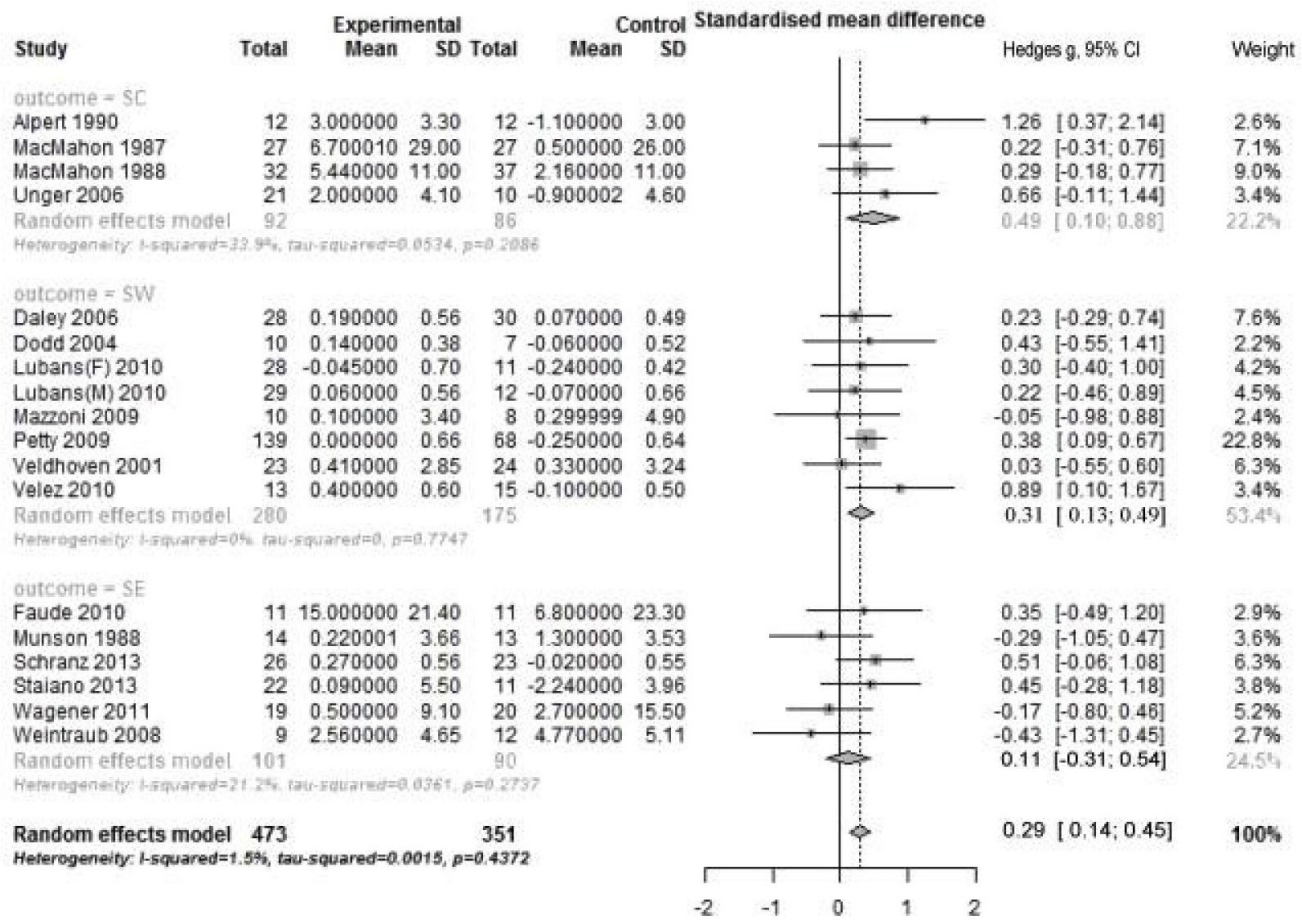
Forest plot of PA alone intervention on self by outcome measure in RCTs. PA: physical activity; SC: self-concept; SW: self-worth; SE: self-esteem; RCTs: randomized controlled trials.

Pooling data from 18 RCTs showed small but significant positive effect for intervention of PA alone (Hedges’ g = 0.29; 95% CI: 0.14 to 0.45; *P* < 0.001) (Fig 2). There was a low heterogeneity across studies (Q _total_ = 9.26; *p* = 0.93; I^2^ = 1.5%), suggesting that the beneficial effect of intervention of PA alone was relatively consistent across RCTs. Stratified analyses by outcome revealed that significant pooled effect sizes were found for intervention of PA alone on SC (Hedges’ g = 0.49, 95% CI: 0.10 to 0.88; p = 0.014) and SW (Hedges’ g = 0.31; 95% CI: 0.13 to 0.49), with no heterogeneity between the subgroup studies. No significant pooled effect size was found on SE.

**Fig 3:**
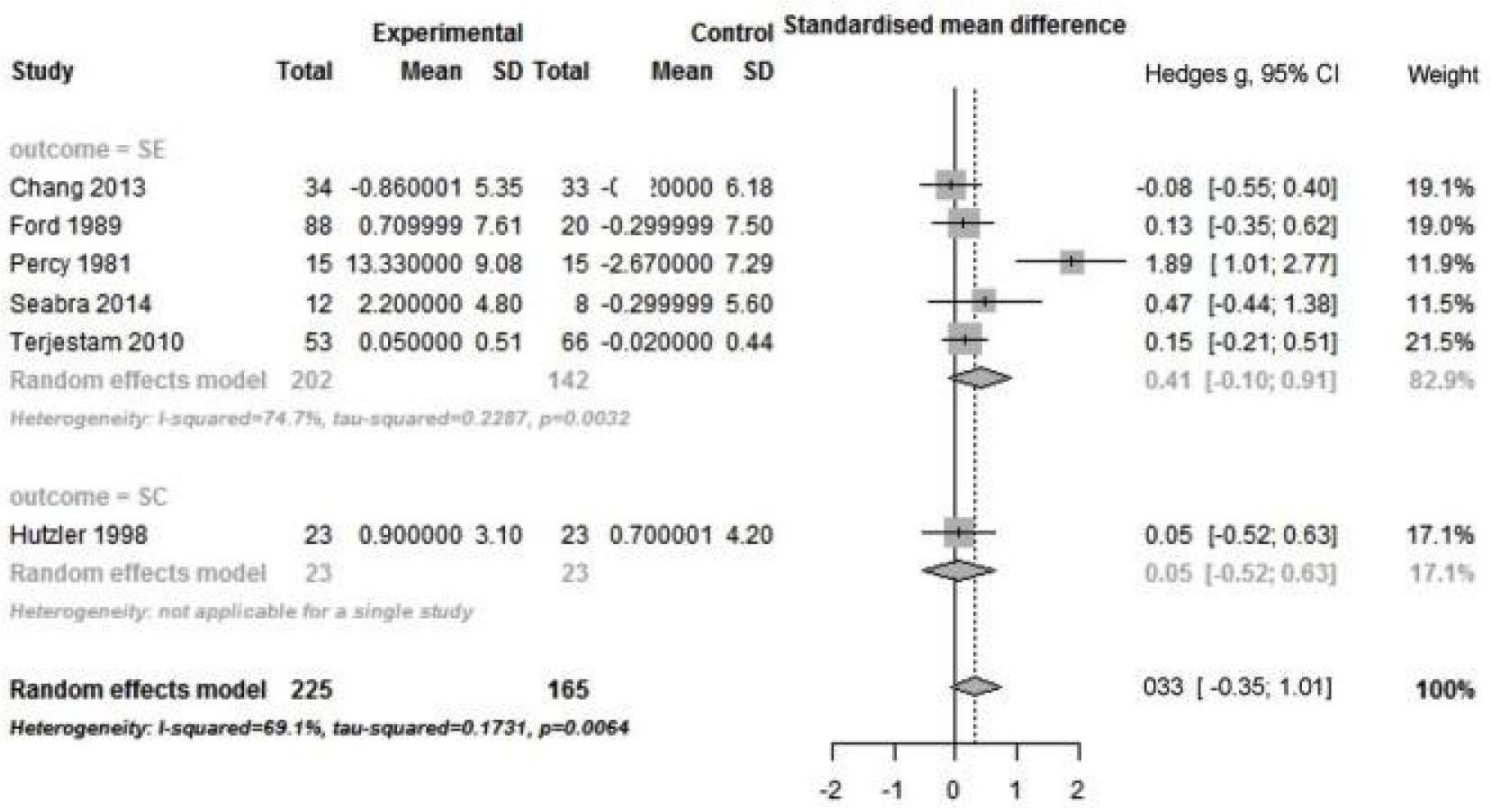
Forest plot of PA alone intervention on self by outcome measure in non-RCTs. PA: physical activity; SE: self-esteem; SC: self-concept; non-RCTs: non-randomized controlled trials.

Based on 6 non-RCTs, no significant pooled effect size was found on general self outcome (Hedges’ g = 0.33; 95%CI: -0.35 to 1.01; p = 0.27) (Fig 3). There was high heterogeneity between studies (Q _total_ = 35.19; *p* < 0.001; I^2^ = 69.1%). Stratified analysis showed that no significant pooled effect size was found on SE. However, high heterogeneity was detected across studies (Q _total_ = 15.84; *p* = 0.003; I^2^ = 74.7%). Limited number of studies precluded stratified analyses for SC and SW.

### Meta-analysis of studies with intervention of PA combined with other strategies

**Fig 4:**
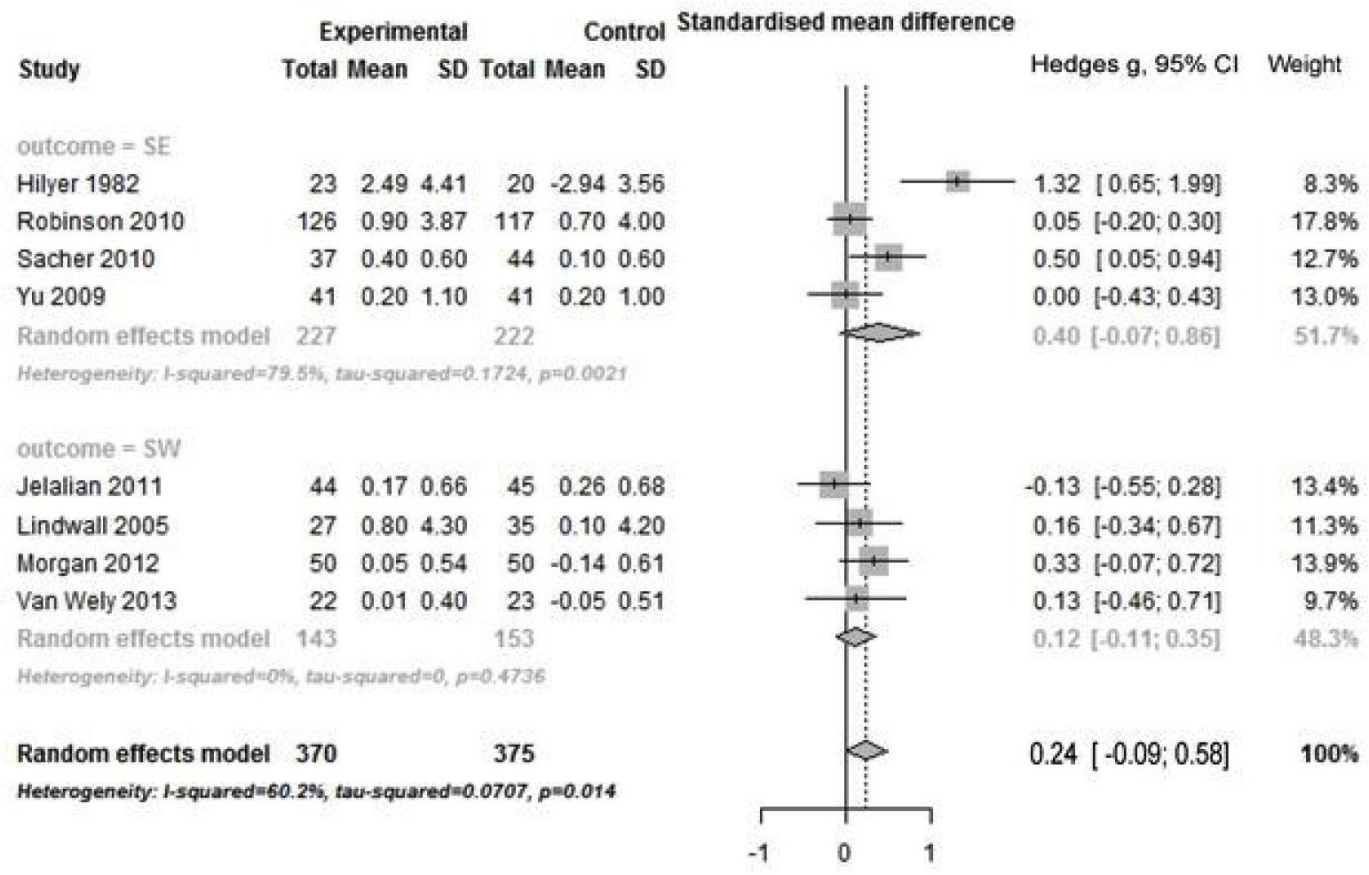
Forest plot of PA combined other intervention on self by outcome measure in RCTs. PA: physical activity; SE: self-esteem; SW: self-worth; RCTs: randomized controlled trials.

**Fig 5:**
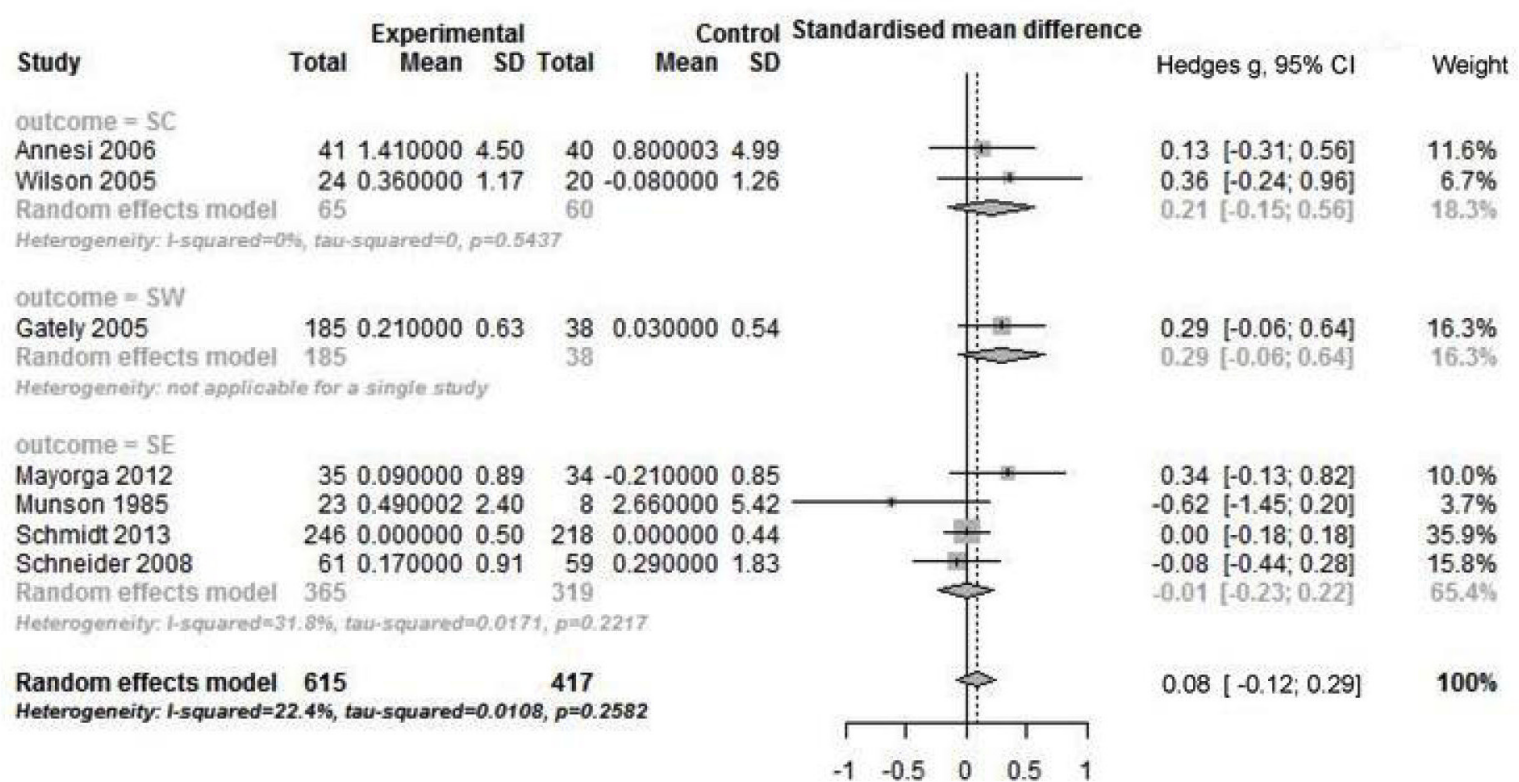
Forest plot of PA combined other intervention on self by outcome measure in non-RCTs. PA: physical activity; SC: self-concept; SW: self-worth; SE: self-esteem; non-RCTs: non-randomized controlled trials.

There was no significant pooled effect size for intervention of PA combined with other strategies on general self outcome. High heterogeneity was found in 8 RCTs (Q _total_ = 7; *p* = 0.05; I^2^ = 60.2%), but not in 7 non-RCTs (Figs 4 and 5). With regards to subtype of outcome, no significant pooled effect size was found in any subgroup, regardless of type of study design. Only for SE outcome, there was high heterogeneity across RCTs (Q _total_ = 14.65; *p* = 0.002; I^2^ = 79.5%).

### Meta-regression analysis

For RCTs, the associations between general self outcome and PA intervention were not substantially altered by intensity of intervention (minutes/session), frequency of intervention (times/week), length of intervention (weeks), participant type (normal vs non-normal), and study quality (low vs high). However, there was a significant association between intervention effect sizes and settings (school, gymnasium, clinic, detention facility, family, or others). Specifically, there was a stronger association with school/gymnasium-based settings than with other settings (regression coefficient β = 0.31, 95% CI: 0.07 to 0.55; p = 0.01) (Table 3). In multivariate meta-regression analysis, after adjusting for potential confounders, the association of intervention effect sizes with settings persisted (β = 0.31, 95% CI: 0.03 to 0.64; p = 0.07).

**Table 3:** Meta- regression analysis.

Variables	Univariate	Multivariate
	Β (95%CI), p	Β (95%CI), p
**RCT study design**		
Quality (ref: ≥5 vs <5)	0.09 (-0.19 to 0.38), 0.916	
Intensity of intervention (min/d)	-0.002 (-0.008, 0.003), 0.39	
Frequency of intervention (times/wk)	0.06 (-0.05 to 0.16), 0.27	
Length of intervention (wks) (continuous)	-0.002 (-0.01 to 0.01), 0.69	
Sample type (ref: non-normal vs normal)	0.10 (-0.27 to 0.46), 0.60	
Setting of intervention (ref: clinc/detention facility/family vs school/gymnasium)	0.31 (0.07 to 0.55), 0.013	0.31 (0.03 to 0.55), 0.068
**Non-RCT study design**		
Quality (ref: ≥6 vs <6)	-0.07 (-0.57 to 0.42), 0.75	
Intensity of intervention (min/d)	0.00 (-0.01 to 0.01), 0.86	
Frequency of intervention (times/wk)	0.03 (-0.14 to 0.19), 0.74	
Length of intervention (wks) (continuous)	-0.01 (-0.03 to 0.01), 0.39	
Sample type (ref: non-normal vs normal)	0.15 (-0.36 to 0.67), 0.53	
Setting of intervention (ref: clinc/detention facility/family vs school/gymnasium)	-0.12 (-0.84 to 0.60), 0.73	

B, regression coefficient; RCT, randomized controlled trial; Non-RCT, non-randomized controlled trial; Ref, reference category.

No significant association was found between effect sizes and intensity of intervention, frequency of intervention, length of intervention, participant type, and study quality in RCTs, as well as all assessed factors in non-RCTs.

### Sensitivity analysis and publication bias analysis

For non-RCTs with intervention of PA alone, sensitivity analysis revealed that heterogeneity between studies was mainly caused by one study conducted by Percy et al[51]. After we omit this study from the analysis, there was no significant heterogeneity for general self (I^2^ from 69.1% to 0%), or SE (I^2^ from 74.7% to 0%) (S1 Fig). Exclusion of this study from the analysis did not substantially alter the overall effect size. For RCTs with intervention of PA combined with other strategies, after excluding a study conducted by Hilyer et al.[26], there was no significant heterogeneity for general self (I^2^ from 60.2% to 0%), or SE (I^2^ from 79.5% to 39.8%)(S2 Fig). Similarly, exclusion of this study from the analysis did not substantially alter the overall effect size.

According to the funnel plot (S3 Fig), there was no asymmetry for RCTs with intervention of PA alone. Begg’s and Egger’s tests revealed no significant publication bias (Begg’s test: *p* = 0.68; Egger’s test: *p* = 0.87). Visual inspection of the funnel plots for non-RCTs with intervention of PA alone and both types of study designs with intervention of PA combined with other strategies showed weak indication of publication bias (S4–S6 Figs), while the Begg’s and Egger’s tests did not suggest significant publication bias (Table 4).

**Table 4:** Meta-analysis of physical activity intervention and self-outcome.

			Pooled effect size	Heterogeneity	Publication bias
Comparison	No	Sample size	SMD (95%CI), P value	Q, P value	P value (Begg’s, Egger’s)
**PA alone intervention**					
RCT	18	824	0.29 (0.14 to 0.45), 0.001	9.26 (0.93)	0.68, 0.87
Non-RCT	6	390	0.33 (-0.35 to 1.01), 0.27	35.19 (<0.001)	0.19, 0.15
**PA combined intervention**					
RCT	8	745	0.24 (-0.09 to 0.58), 0.13	7 (0.05)	0.14, 0.16
Non-RCT	7	1032	0.08 (-0.12 to 0.29), 0.36	4.39 (0.62)	0.88, 0.77

SMD, standardized mean difference; PA, physical activity; RCT, randomized controlled trial; Non-RCT, non-randomized controlled trial.

## Discussion

Results of this meta-analysis suggest that intervention of PA alone is an effective method to improve SW and SC in juveniles, although the effect sizes were small in magnitude. The lack of publication bias and very low heterogeneity in RCTs evaluating intervention of PA alone and non-RCTs evaluating intervention of PA combined with other strategies suggest that our results were relatively robust. However, caution is required in interpretation of effects of intervention of PA alone in non-RCTs and intervention of PA combined with other strategies in RCTs, since there were significant heterogeneities across studies.

We further identified that, one non-RCT [51] with intervention of PA alone with small sample size, as well as one RCT with intervention of PA combined with other strategies [26] contributed to the majority of the observed heterogeneity. However, sensitivity analyses showed that omitting these studies did not substantially alter the pooled effect sizes.

Meta-regression analysis revealed that the associations between PA intervention and self outcome could be altered by setting of intervention in RCTs. There were stronger effects in studies with school-based or gymnasium-based interventions compared with studies with family-based, clinic-based, and detention facility-based interventions. One possible explanation is that schools and gymnasiums are places where services are both mandated and free for juveniles[35, 52]. There is also a possibility that studies focused on other settings are few, making the power to be insufficient[53, 54]. More studies with PA interventions focusing on these settings are warranted to clarify this issue.

Our results confirm the findings of the meta-analysis conducted by Ekland and colleagues[34]. Our findings were also consistent with those of the meta-analysis conducted by Ahn et al. in RCTs, but varied widely regarding to non-RCT study analysis [35]. This might be due to that the non-RCT studies in their analysis included those with within-subject design (i.e., pretest-posttest single group design) and between-subject design (i.e., posttest-only-control group design), while only independent-groups design (pretest-posttest-control group design) was included in the current meta-analysis. In contrast to a previous analysis by Ekland et al, we did not find any beneficial effect of intervention of PA combined with other strategies on self outcomes, regardless of study design. Considering the baseline difference between the treatment group and control group, our analysis pooled effect sizes by computing effect sizes in each treatment condition and then subtracting the effect size of the control group from the intervention group. However, previous studies only considered post-intervention mean difference when pooling effect sizes, which may induce statistical errors when there was significant difference with regard to the outcome at baseline for relevant studies [55, 56].

For RCTs with intervention of PA alone, our results were consistent with the previous studies[34, 35]. However, the effect sizes on different outcome measurements were not equal. For example, Ekeland et al. found a moderate effect size of intervention of PA alone on SE, comparing with minor effect sizes on general self outcome and SW, a moderate effect size on SC, and no significant effect size on SE in our meta-analysis. This may be due to the different outcome definitions used between the two analyses. The definitions of our outcomes were based on the final measurements of outcomes for each study rather than the planned outcomes, since there are a wide range of different definitions for SE and SC. Actually, some studies used SC or SW questionnaires to measure SE. For example, one study[13] conducted before 1990 was considered to have assessed SE outcome in Ekeland et al’s analysis, however, we coded the outcome as SC since it used a “Thomas Self-Concept Values Test”. Compared with the analyses conducted by Ekeland et al. and Ahn et al., our study performed additional analyses for evaluating the effect sizes of PA intervention on different outcomes according to outcome measurements. Our results showed that these concepts may not be completely equal for representing individual self. Therefore, the use of terminology in the research questions of interest should be cautious, and further research is warranted. Moreover, small sample sizes of included studies in the subgroup of SE outcome may influence the effect size of intervention of PA alone. Thus, studies with larger sample sizes are needed to further clarify this issue.

It is worth to note that we found no beneficial effect of PA intervention on self outcomes in non-RCTs, regardless of study design and outcome measurements. This is different from findings of the meta-analysis conducted by Ahn et al., which showed a relatively large effect size (Hedgs’ g = 0.78) of PA intervention on SE. This may be due in part to a wider range of non-RCTs study designs included in Ahn et al’s meta-analysis, such as within-subject design (i.e., pretest-posttest single group design) and between-subject design (i.e., posttest-only-control group design)[35]. Moreover, different methods of synthesis of effect sizes may contribute to the discrepancy. However, we need to note that there was no significant beneficial effect of PA on SC in Ahn et al.’s analysis for non-RCTs. We supposed that non-RCT design might lead to more bias on effect sizes. For example, the potentially different conditions between the treatment group and the control group may induce the difference. Also the effect of potential baseline difference cannot be excluded. These may partially lead to the high heterogeneity in the analysis of non-RCTs.

### Strengths and limitations of the review

The present meta-analysis was based on prospective RCTs and non-RCTs involving multiple types of juvenile. The involved sample size was relatively large. Analyses for subtype of PA intervention design (PA alone, or PA combined with other strategies), in combination with subtype of study design (RCTs, or non-RCTs) and outcome measure (SE, SC, or SW) were conducted. Moreover, we performed several sensitivity analyses and meta-regression to explore the potential sources of heterogeneity and moderators.

However, there were several limitations. First, a wide range of different contents in PA intervention made it difficult to identify intervention type. Studies with intervention of PA alone may be classified as with the intervention of PA combined with other strategies. There is a similar difficulty for identifying study design, in that some studies which were claimed as RCTs were actually non-RCTs. In the current study we classified them as non-RCTs if we could not find sufficient information indicating that RCT design was used. This may lead to incorrect categories. Furthermore, there were differences in measurement of self outcome across studies. These factors may affect the pooled effect sizes. The limited number of studies in some subtype groups, such as different sample type, setting of PA intervention, and outcome measurement, precluded further subgroup analyses or meta regression analysis.

## Conclusion

In conclusion, this meta-analysis provides further evidence that intervention of PA alone plays a role in improving SW and SC in children and adolescents. The results support current recommendations for increasing PA to promote physical and mental health. Results of this review also reveal that setting of PA intervention is potentially important to affect the effect of PA intervention on self outcome, and there is stronger association with school-based and gymnasium-based intervention compared with other settings. However, the inherent limitations of imbalanced sample type, outcome measurement, and setting of PA intervention within included studies prevent us from attaining definitive conclusions. Future well-designed RCTs and multiple levels of PA intervention targeting on various sample types are warranted to validate the findings of the current analysis.

## Practical Implications

For children and adolescents, intervention of PA alone is beneficial to SW and SC. Moreover, school-based and gymnasium-based PA interventions may exert stronger effects on developing SE and SC.

## Supporting Information

S1 Fig Influence analysis of PA alone intervention on SE in non-RCTs. (PDF)

S2 Fig Influence analysis of PA combined intervention on SE in RCTs. (PDF)

S3 Fig Funnel plot of PA alone intervention and self outcome in RCTs. (PDF)

S4 Fig Funnel plot of PA alone intervention and self outcome in non-RCTs. (PDF)

S5 Fig Funnel plot of PA combined intervention and self outcome in RCTs. (PDF)

S6 Fig Funnel plot of PA combined intervention and self outcome in non-RCTs. (PDF)

S1 File PRISMA checklist and the search strategy. (PDF)
