# Supplementary figures and images for: How Does Physical Activity Intervention Improve Self-Esteem and Self-Concept in Children and Adolescents? Evidence from a Meta-Analysis
Source: PLoS One. 2015 Aug 4;10(8):e0134804. doi: 10.1371/journal.pone.0134804 (PMC4524727; doi:10.1371/journal.pone.0134804)

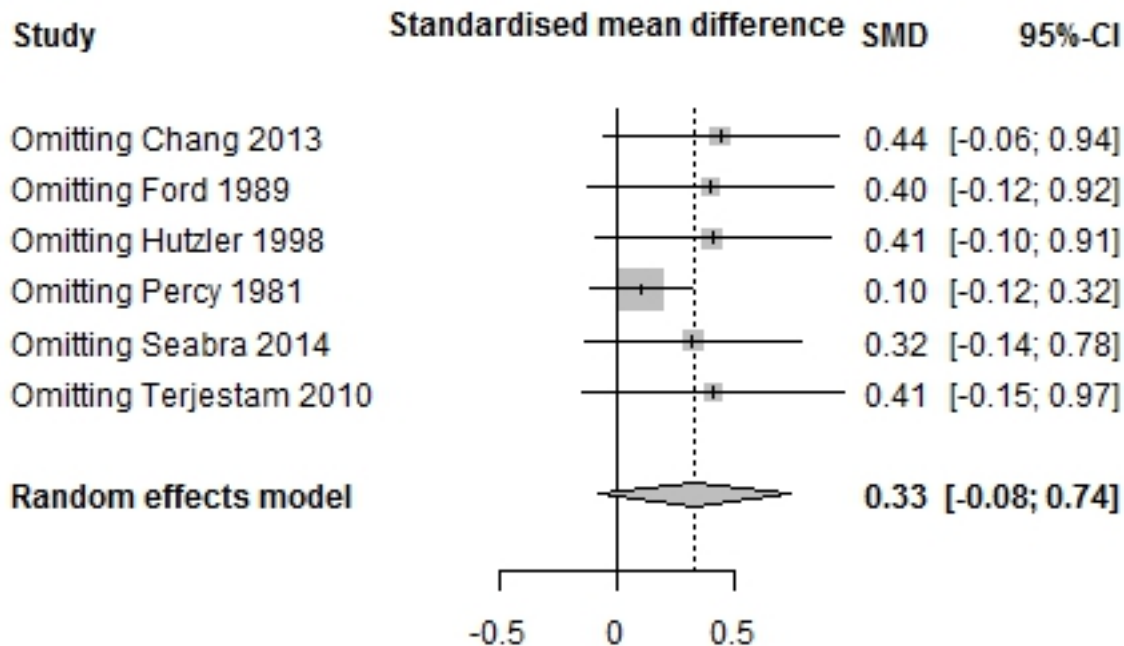

Supplement: S1 Fig — (PDF) [file pone.0134804.s001.pdf]

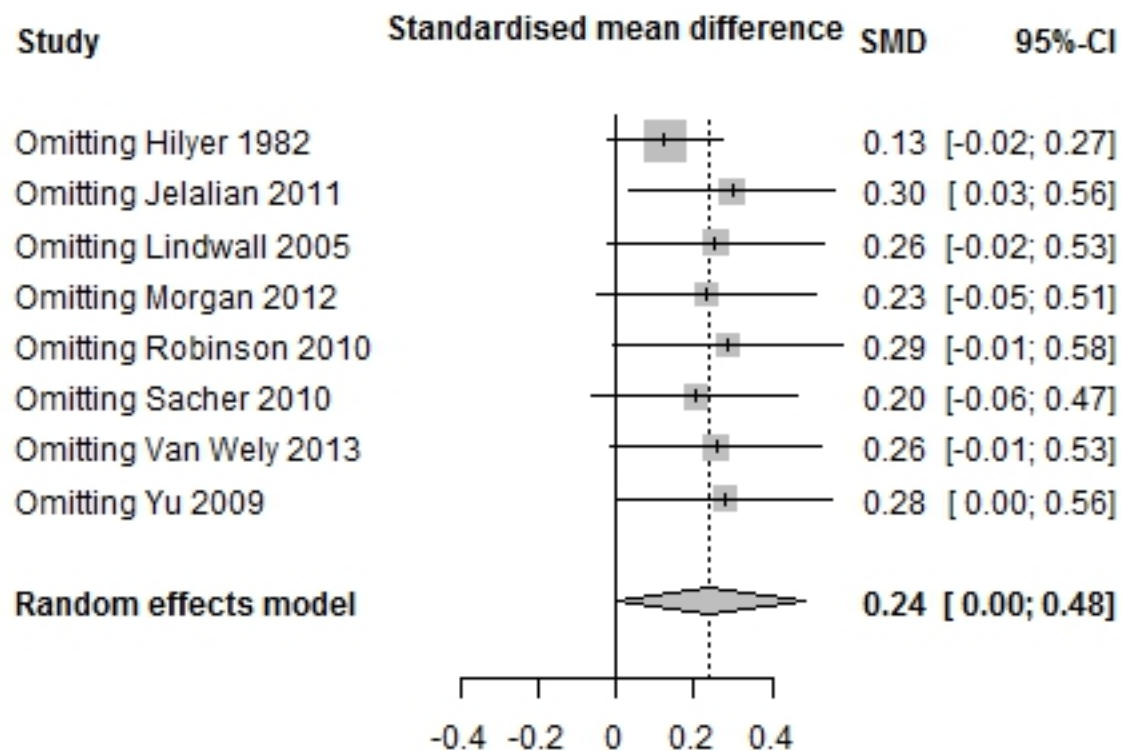

Supplement: S2 Fig — (PDF) [file pone.0134804.s002.pdf]

Begg's funnel plot with pseudo 95% confidence limits

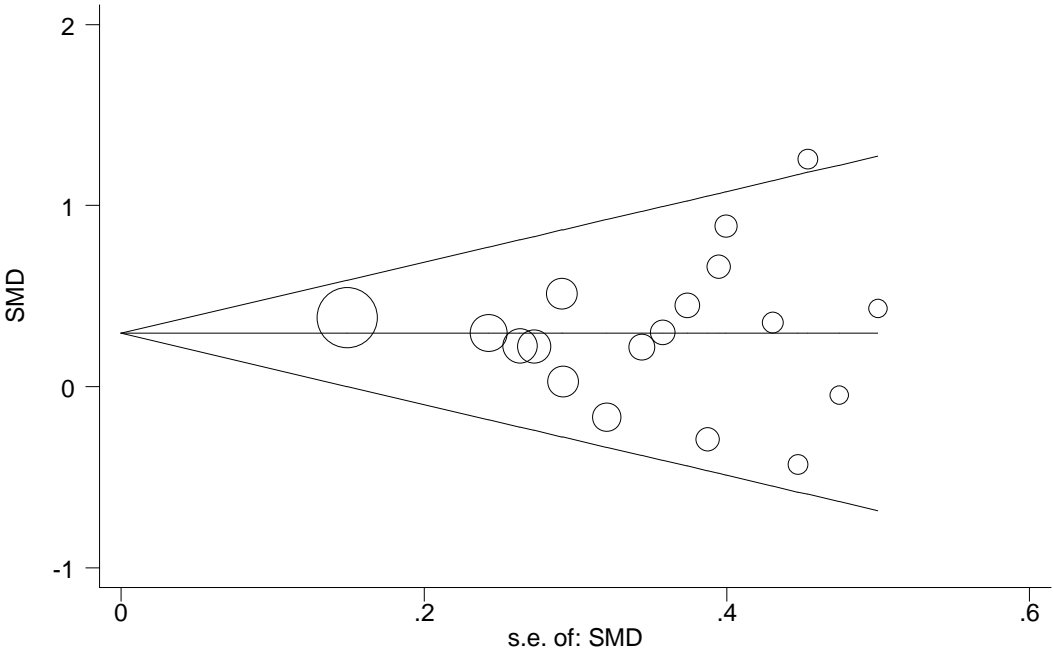

Supplement: S3 Fig — (PDF) [file pone.0134804.s003.pdf]

Begg's funnel plot with pseudo 95% confidence limits

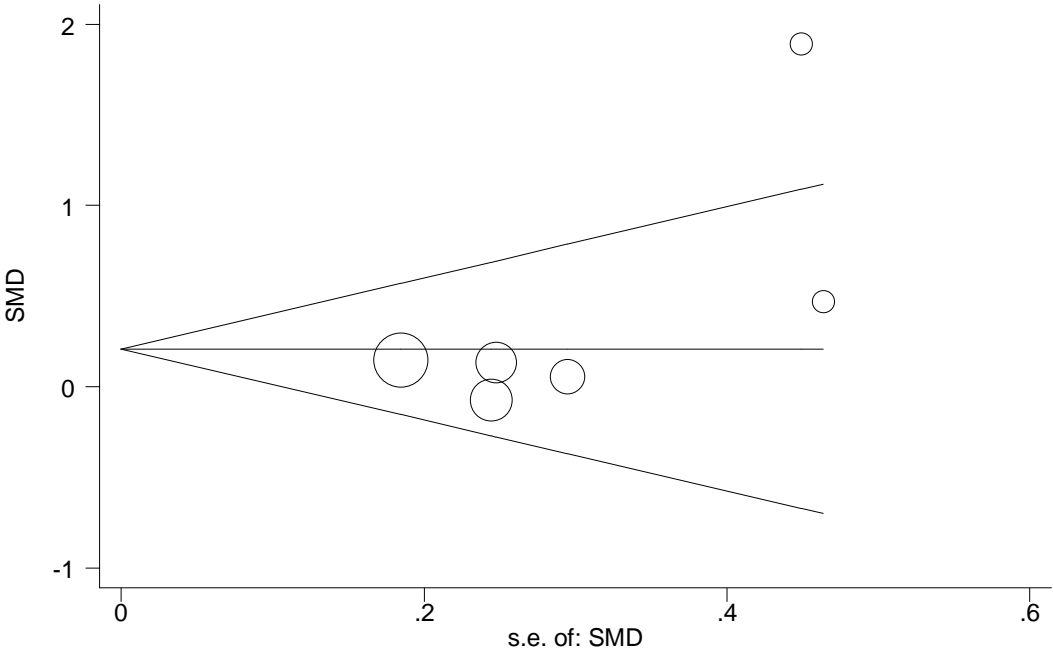

Supplement: S4 Fig — (PDF) [file pone.0134804.s004.pdf]

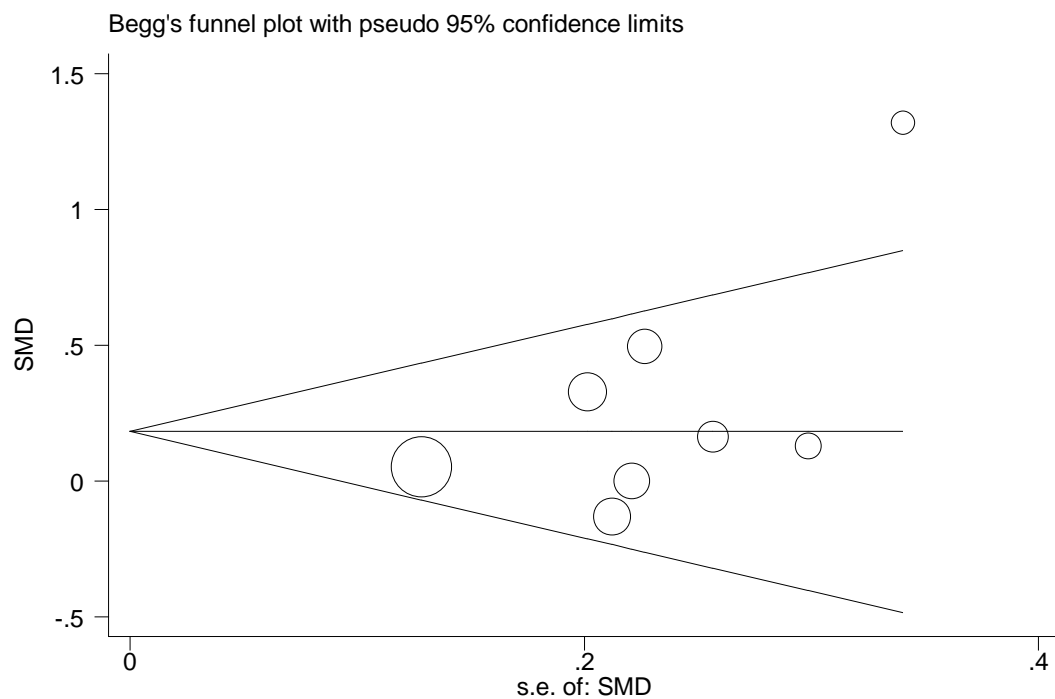

Supplement: S5 Fig — (PDF) [file pone.0134804.s005.pdf]

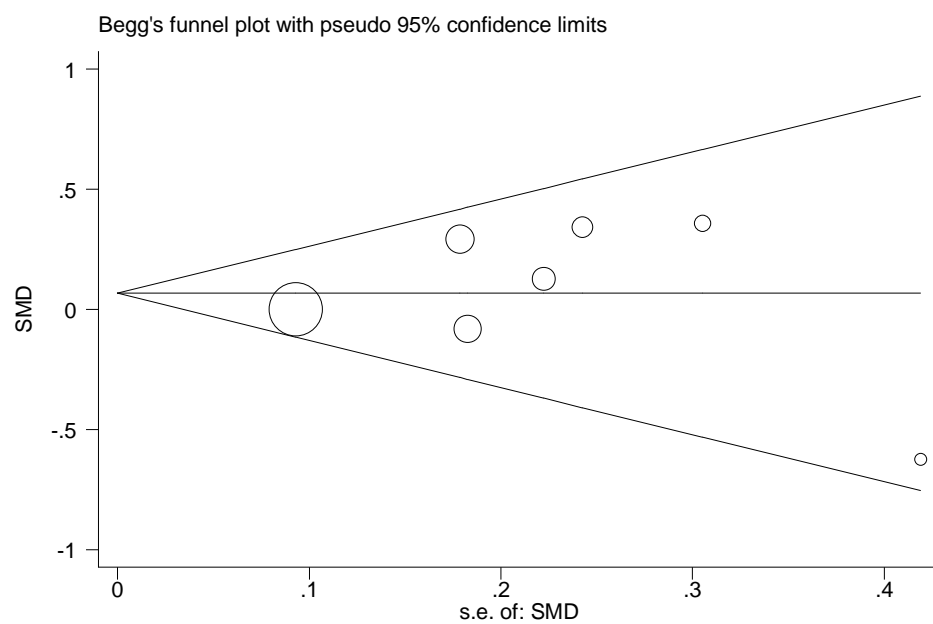

Supplement: S6 Fig — (PDF) [file pone.0134804.s006.pdf]
